# Development of a Norway rat hepacivirus reporter for high-throughput quantification of neutralizing antibodies

**DOI:** 10.1128/jvi.01943-25

**Published:** 2026-03-31

**Authors:** Matthew J. Kennedy, Raphael Wolfisberg, Emma A. Lundsgaard, Caroline E. Thorselius, Laura Collignon, Louise Nielsen, Kenn Holmbeck, Jens Bukh, Troels K. H. Scheel

**Affiliations:** 1 Copenhagen Hepatitis C Program (CO-HEP), Department of Infectious Diseases, Hvidovre Hospital and Department of Immunology and Microbiology, University of Copenhagen 4321, Copenhagen, Denmark; Wake Forest University School of Medicine, Winston-Salem, North Carolina, USA

**Keywords:** virus-host interactions, neutralizing antibodies, reporter virus, animal model, hepatitis C virus, hepacivirus

## Abstract

Hepatitis C virus (HCV) remains a major global health challenge, with vaccine research hindered by a lack of robust immunocompetent animal models. Infection with the HCV-related Norway rat hepacivirus 1 (NrHV) in rats or mice shares HCV-defining characteristics, including chronicity, delayed immune responses, and liver disease, thereby providing immunocompetent small animal models for immune responses and pathology. Infectious NrHV cell culture systems allow studies of host factors and neutralizing antibodies; however, quantification relies on laborious manual counting of infected cells. To permit high-throughput quantification, we engineered culture-adapted NrHV to encode firefly luciferase (FLuc), green fluorescent protein (GFP), or the 11 amino acid HiBiT tag at sites corresponding to those permissive for HCV. While FLuc and GFP constructs were not viable, a reporter with a HiBiT insertion in the NS5A domain III was replication competent and produced infectious virus in rat hepatoma cells. Two mutations in NS5A adapted this virus to levels comparable to wild type, enabling robust luciferase readouts. The NrHV-HiBiT-NS5A reporter was stable, viable in rats, and permitted quantification of luciferase from liver homogenates. Luciferase-based quantification of viral infection after antibody neutralization correlated well with manual quantification. High-throughput luciferase quantification was also used to confirm dependency on the known host factors SR-BI and miR-122 and to assess sensitivity to antivirals. Finally, sensitivity to a specific inhibitor suggested that cyclophilin A, similarly to HCV, may be a host factor for NrHV. This novel reporter system could accelerate characterization of the NrHV model as well as ongoing vaccine studies for HCV.

**IMPORTANCE:**

Direct-acting antiviral therapy provides an effective cure for chronic hepatitis C virus (HCV) infection, but the infection burden remains high due to cost and availability of therapy and low diagnosis rates. A vaccine, therefore, is critical for global control of viral transmission; however, vaccine development is constrained by a lack of robust immunocompetent animal models. Norway rat hepacivirus 1 (NrHV) infection of rats and mice serves as an effective small animal model for HCV, permitting evaluation of vaccine platforms and candidates. Neutralizing antibody responses are considered crucial for protection and can be quantified using the NrHV cell culture system, however, only through laborious low-throughput methods. We here present an NrHV reporter virus allowing high-throughput quantification of antibody neutralization, virus-host interactions, and antiviral efficacy via luciferase readout. This reporter will facilitate characterization of NrHV and support ongoing vaccine research with the potential for global control of HCV.

## INTRODUCTION

Hepatitis C virus (HCV) chronically infects ~50 million people worldwide, leading to increased risk of liver cirrhosis and hepatocellular carcinoma (1). While direct-acting antiviral (DAA) therapy achieves cure rates of >90%, this remains insufficient for global control of the virus due to low diagnosis rates and the high cost and low accessibility of DAA therapy in low- and middle-income countries with the highest prevalence of HCV infection (2, 3). Accordingly, a vaccine is required to achieve global control of HCV (4).

Vaccine research for HCV, which naturally only infects humans and experimentally also chimpanzees, suffers from a lack of immunocompetent animal models for challenge studies. While small animal models, such as entry factor transgenic or human liver-chimeric mice, are available, they are challenged by a lack of robust infection or blunted immune responses, respectively, making them unsuited for use in studies of vaccines and immunopathology (5). For years, the closest known HCV relative was GB virus B (GBV-B), a virus infecting New World monkeys (6). Deep sequencing efforts have more recently resulted in the expansion of our knowledge of the hepacivirus genus, revealing HCV-related viruses in a variety of species, including rodents, bats, monkeys, horses, and cattle (7, 8). Among these, Norway rat hepacivirus 1 (NrHV; rodent hepacivirus of *Rattus norvecigus*, RHV-rn1) is of particular interest as an HCV model given its tropism for small rodent laboratory animals.

Similar to HCV, NrHV is a positive-stranded RNA virus of ~9.6 kb, with a single open reading frame (ORF) flanked by 5′ and 3′ untranslated regions (UTRs). Translation is driven from an internal ribosomal entry site (IRES) in the 5′ UTR and leads to expression of a single polyprotein that is predicted to be processed into three structural (Core, E1, and E2) and seven non-structural (p7, NS2, NS3, NS4A, NS4B, NS5A, and NS5B) proteins. NrHV furthermore relies on the liver-specific microRNA, miR-122, for replication (9, 10). NrHV entry into hepatocytes depends on rat orthologs of the HCV host factors scavenger receptor class B type 1 (SR-BI), CD81, and occludin (OCLN), as well as claudin-3 (CLDN3), although there may be redundancy in the use of some of these factors (11, 12). NrHV leads to chronic, high titer, hepatotropic infection in rats, inducing a delayed development of specific T-cell and neutralizing antibody (nAb) responses, and with the capacity to induce liver damage (11, 13, 14). With hallmarks of infection comparable to those of HCV, NrHV is well-suited as an immunocompetent model for the study of hepaciviral infection, pathology, and immune responses. NrHV also infects mice, in which infection is acutely resolved within 2–3 weeks. Infection can be extended to 5 weeks through mouse-adaptive mutations or even to chronicity via depletion of CD4^+^ T cells preceding infection (10, 15). The development of the NrHV replicon and infectious cell culture systems in rat hepatocytes permitted the study of the full viral life cycle *in vitro*, providing means to study virus-host interactions and to assess and quantify nAbs generated from infected animals (9, 11). The variety of *in vivo* and *in vitro* systems available for NrHV thus makes it a compelling model for essential research into pathology and immune responses. While viral epitopes and immune responses from NrHV studies may not directly transfer to HCV, the ability to test putative vaccine candidates and combinations thereof in an immunocompetent host amenable to challenge provides an important tool in the ongoing quest for an HCV vaccine (7).

The current culture systems allow studies of host factors, antiviral compounds, and NrHV targeting nAbs (11, 15–17); however, they rely on laborious manual quantification of focus-forming units (FFUs) following antigen staining. An NrHV reporter virus would permit high-throughput analysis of nAbs and host factors with reduced inter-operator variation. We here present the development of a viable and stable NrHV reporter virus by the insertion of the HiBiT tag (18) and accompanying adaptive mutations into the NS5A domain III. Luciferase signal from the HiBiT detection system led to quantification of viral infection after antibody neutralization, host factor inhibition, or antiviral compounds comparable to manual FFU counts, but in a high-throughput manner. This provides a much-needed tool to expedite HCV-relevant vaccine research using the NrHV model. In addition, this reporter could aid further characterization of NrHV, increasing our knowledge of hepacivirus evolution and the utility of NrHV as a model.

## RESULTS

### Low permissiveness for insertion of reporter genes into the NrHV ORF

**Fig 1:**
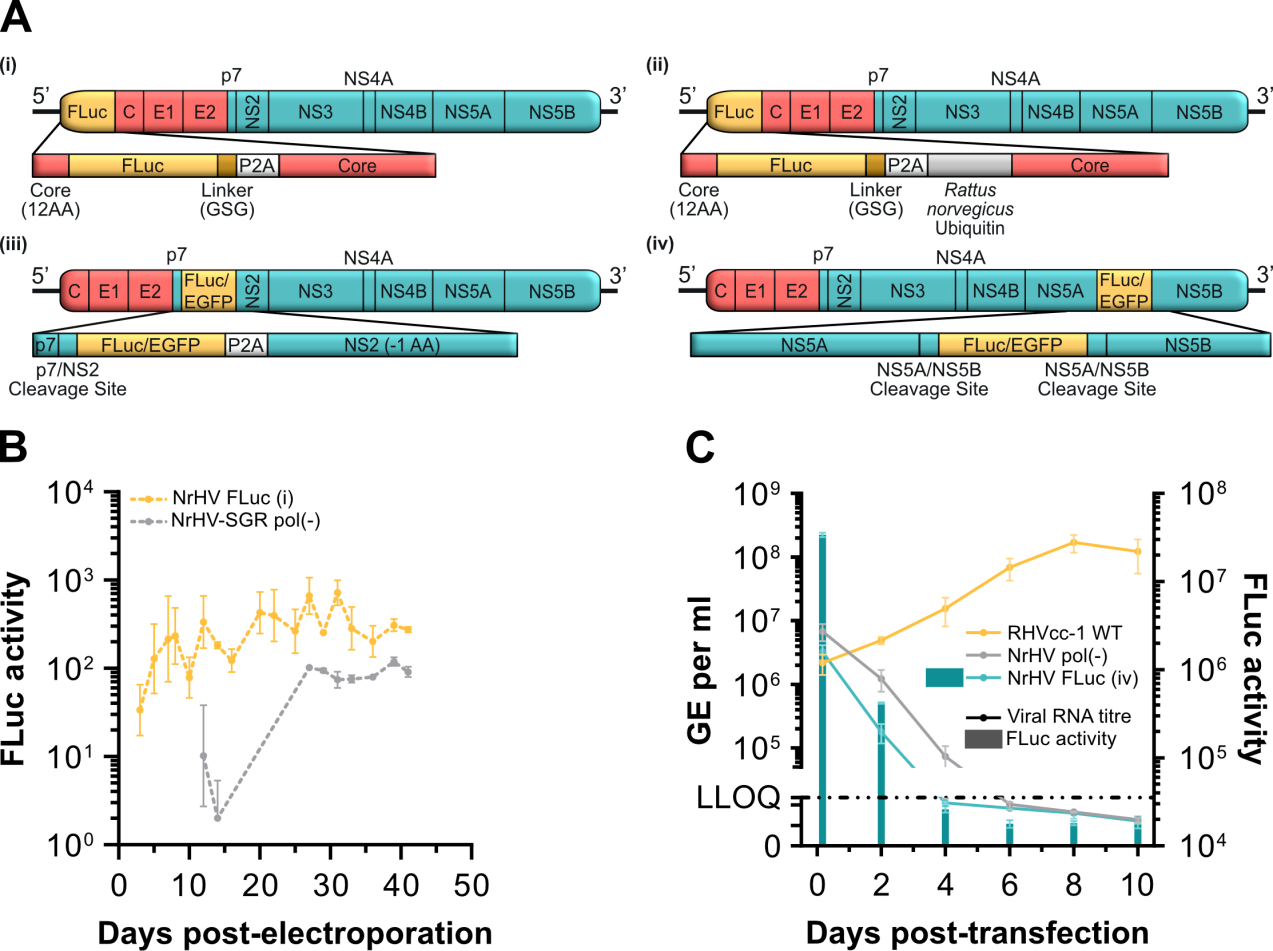
Low permissiveness for insertion of reporter genes into the NrHV ORF. (**A**) Schematic representations of four NrHV reporter constructs with insertions at the following sites: (i) FLuc inserted downstream of the first 12 AA of core and upstream of a GSG linker, a P2A sequence, and the full core protein; (ii) the same design as reporter (i) but with *Rattus norvegicus* ubiquitin inserted between the P2A sequence and core; (iii) either FLuc or EGFP inserted between p7 and NS2, downstream of the p7/NS2 cleavage site and upstream of a P2A sequence and NS2 lacking the first AA; (iv) either FLuc or EGFP inserted between duplicated NS5A/NS5B cleavage sites. (**B**) FLuc activity for NrHV-FLuc (i) and a replication-deficient NrHV sub-genomic FLuc replicon over time after electroporation of IVT RNA into NrHV permissive McA-RH7777.hi rat hepatoma cells. Data points represent means ± standard deviation (SD) from triplicates for days 1–7, and duplicates thereafter. (**C**) Comparison of FLuc activity and RNA genome equivalents (GE) per mL of supernatant over time after transfection of IVT RNA of RHVcc-1, reporter NrHV-FLuc (iv), or a replication-deficient NrHV sub-genomic FLuc replicon into NrHV permissive McA-RH7777.hi rat hepatocytes. The lower limit of quantification (LLOQ) for RT-qPCR was 5 × 10^4^ GE/mL. Solid lines indicate RNA titers, while bars represent FLuc activity. Data points represent means ± SD from triplicates. All replicates for individual data points are technical replicates of a single transfection or electroporation.

To generate an NrHV reporter virus, we first inserted a UpA/CpG-optimized firefly luciferase (FLuc) gene (19) into the ORF of RHVcc-1, a cell culture-adapted clone of the RHV-rn1 strain (11). Insertion sites were selected based on sites permissive for HCV: (i) at the N-terminus of core, downstream of the first 12 amino acids (AAs) and upstream of a GSG linker, a P2A sequence to allow for cleavage, and the complete core protein (20); (ii) at the same position, but followed by rat ubiquitin downstream of P2A, allowing cleavage to generate the native N-terminus of core; (iii) between p7 and NS2, with FLuc followed by P2A and NS2 lacking the first AA (21); and (iv) between duplicated NS5A/NS5B cleavage sites (22) (Fig. 1A). *In vitro* transcribed (IVT) RNA of these reporter constructs was transfected or electroporated into McA-RH7777.hi rat hepatoma cells with increased permissiveness to NrHV (9), with wild-type (WT) RHVcc-1 as a positive control. Cultures were followed for at least 4 weeks by antigen staining and luciferase measurements. Robust antigen staining or luciferase signal was never observed for NrHV-FLuc (i), (ii), and (iii). Only low luciferase signal was detectable, as exemplified for >40 days post-electroporation of NrHV-FLuc (i) (Fig. 1B). For construct (iv), robust luciferase signal and viral RNA titers were observed early after transfection, but these rapidly declined, comparable to a non-replicating pol(–) control (Fig. 1C). To exclude that observed deficiencies in viral replication were due to elements of the FLuc sequence, we additionally engineered constructs (iii) and (iv) with UpA/CpG-optimized enhanced green fluorescent protein (EGFP). However, no EGFP signal was observed by microscopy during >4 weeks of monitoring following transfection of IVT RNA. Thus, robust replication was not observed for any of the tested reporter constructs.

### Active replication of NrHV with a HiBiT tag inserted into NS2 or NS5A

**Fig 2:**
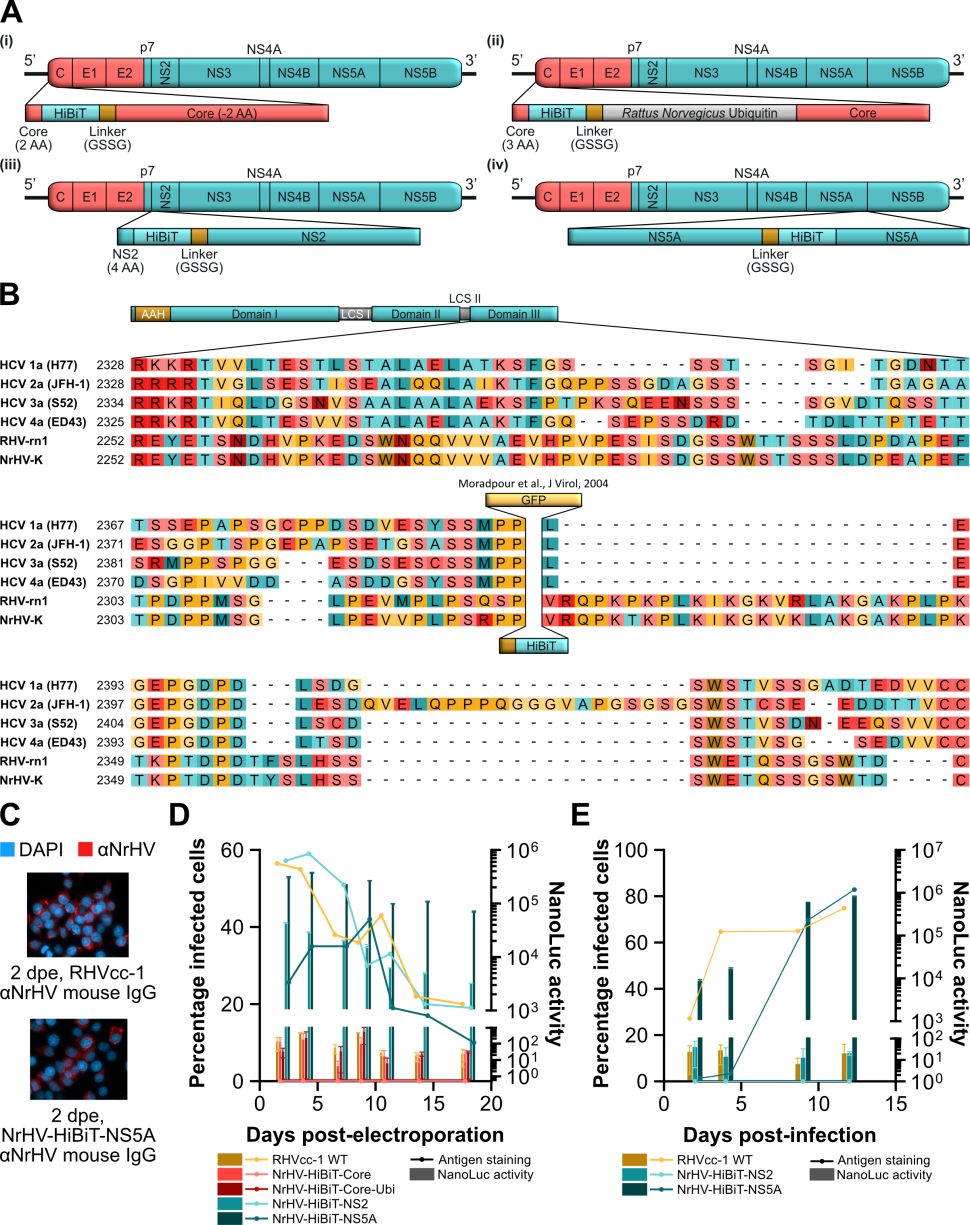
Insertion of the HiBiT tag into NS5A permits replication and infectious virus production. (**A**) Schematic representations of four NrHV reporter constructs with insertions of the HiBiT tag at the following sites: (i) NrHV-HiBiT-Core, at the N-terminus of core following the first 2 AA and upstream of a GSSG linker and core lacking the first 2 AA; (ii) NrHV-HiBiT-Core-Ubi, at the N-terminus of core following the first 3 AA and upstream of a GSSG linker, *Rattus norvegicus* ubiquitin, and the full core protein; (iii) NrHV-HiBiT-NS2, at the N-terminus of NS2 following the first 2 AA of NS2 and upstream of a GSSG linker and the full NS2 protein; and (iv) NrHV-HiBiT-NS5A, within NS5A domain III following AA 2322 and a GSSG linker. (**B**) MAFFT multiple sequence alignment of NS5A of HCV genotype 1a (H77, GenBank accession no. AF009606), HCV genotype 2a (JFH-1, GenBank accession no. AB047639), HCV genotype 3a (S52, GenBank accession no. GU814264), HCV genotype 4a (ED43, GenBank accession no. GU814265), RHV-rn1 (GenBank accession no. KX905133), and NrHV-K (GenBank accession no. PV553238) (27). Shown is the NS5A domain III (based on the domain definition for HCV) with indication of the insertion site of both the HiBiT tag and GFP in a functional HCV reporter (25, 26). Due to the low conservation of the depicted region, different alignment algorithms yielded variation in the resulting alignments. (**C and D**) Electroporation of IVT RNA of RHVcc-1 and HiBiT reporter constructs, as shown in (**A**), into NrHV permissive McA-RH7777.hi rat hepatoma cells: (**C**) imaging of antigen-stained RHVcc-1 and NrHV-HiBiT-NS5A 2 days post-electroporation (dpe), using total IgG from C57BL/6 mice that resolved NrHV infection, and (**D**) time course with solid lines indicating manually enumerated percentage of infected cells and bars representing NanoLuc activity subtracted mean signal from reagent-only control wells. Data points represent individual values for the percentage of infected cells, and means ± SD from duplicates for NanoLuc activity. (**E**) Infection of naïve McA-RH7777.hi rat hepatoma cells with supernatant collected from cells electroporated with RHVcc-1, NrHV-HiBiT-NS2, or NrHV-HiBiT-NS5A IVT RNA in the previous experiment (**D**), 9 days post-electroporation. Data points represent means ± SD from triplicates. All NanoLuc activity was normalized to signal per 70,000 cells. All replicates for individual data points are technical replicates of a single electroporation or infection.

Reasoning that full-length reporter genes may not be readily accommodated within the NrHV genome due to size restrictions, we instead inserted sequence encoding the 11 AA HiBiT tag at four positions in the NrHV ORF. The small HiBiT tag constitutes a functional NanoLuc luciferase enzyme when bound to the larger complementary LgBiT protein, allowing for quantification of HiBiT-tagged viral proteins upon lysis and addition of the LgBiT protein. Due to the requirement of appending the HiBiT tag onto proteins, the selected insertion sites differed but were again based on sites permissive for HCV: (i) after the first 2 AA of core, upstream of a GSSG linker and the remainder of core (23); (ii) after the first 3 AA of core, upstream of a GSSG linker, rat ubiquitin, and the full core protein; (iii) at the N-terminus of NS2, downstream of a duplication of the first 4 AAs of NS2 and upstream of a GSSG linker (24); and (iv) after AA 2322 within NS5A domain III, downstream of a GSSG linker (25, 26) (Fig. 2A). Of note, conservation of NS5A domain III between NrHV and HCV, and even between HCV isolates, is low, challenging identification of the insertion site corresponding to that successfully employed for HCV (Fig. 2B). IVT RNA of these NrHV reporter constructs, and the RHVcc-1 control, was then electroporated into McA-RH7777.hi cells. The infection was followed over time using antigen staining and measurement of NanoLuc luciferase (Fig. 2C and D). No antigen-positive cells and only near-background luciferase signal were detected for the NrHV-HiBiT-Core and NrHV-HiBiT-Core-Ubi constructs. In contrast, NrHV-HiBiT-NS2 and NrHV-HiBiT-NS5A replication was detectable by both luciferase and antigen staining. NrHV-HiBiT-NS2 showed antigen-positive cell counts comparable to the RHVcc-1 WT control, with a luciferase signal that followed this trend. NrHV-HiBiT-NS5A showed reduced antigen-positive cells at early time points but reached WT levels by 7 days post-electroporation. The luciferase signal observed for NrHV-HiBiT-NS5A correlated with the quantification of antigen-positive cells over time. Furthermore, NrHV-HiBiT-NS5A had increasingly higher luciferase signal compared to NrHV-HiBiT-NS2, with the difference increasing from ~7-fold to >21-fold higher signal from 2 to 18 days post-electroporation.

To assess the production of infectious particles, supernatants from NrHV-HiBiT-NS2- and NrHV-HiBiT-NS5A-electroporated cells (9 days post-electroporation) were passaged onto naïve McA-RH7777.hi cells. No infection was observed for NrHV-HiBiT-NS2, while NrHV-HiBiT-NS5A exhibited a delayed spread of infection, eventually spreading to a fraction of cells comparable to the WT at 9 days post-infection (Fig. 2E). The luciferase signal for NrHV-HiBiT-NS5A again closely followed quantification of percentage of infected cells. Taken together, these results suggested that NrHV-HiBiT-NS5A is a functional reporter virus.

### Mutations in NS5A adapt NrHV-HiBiT-NS5A to cell culture

**Fig 3:**
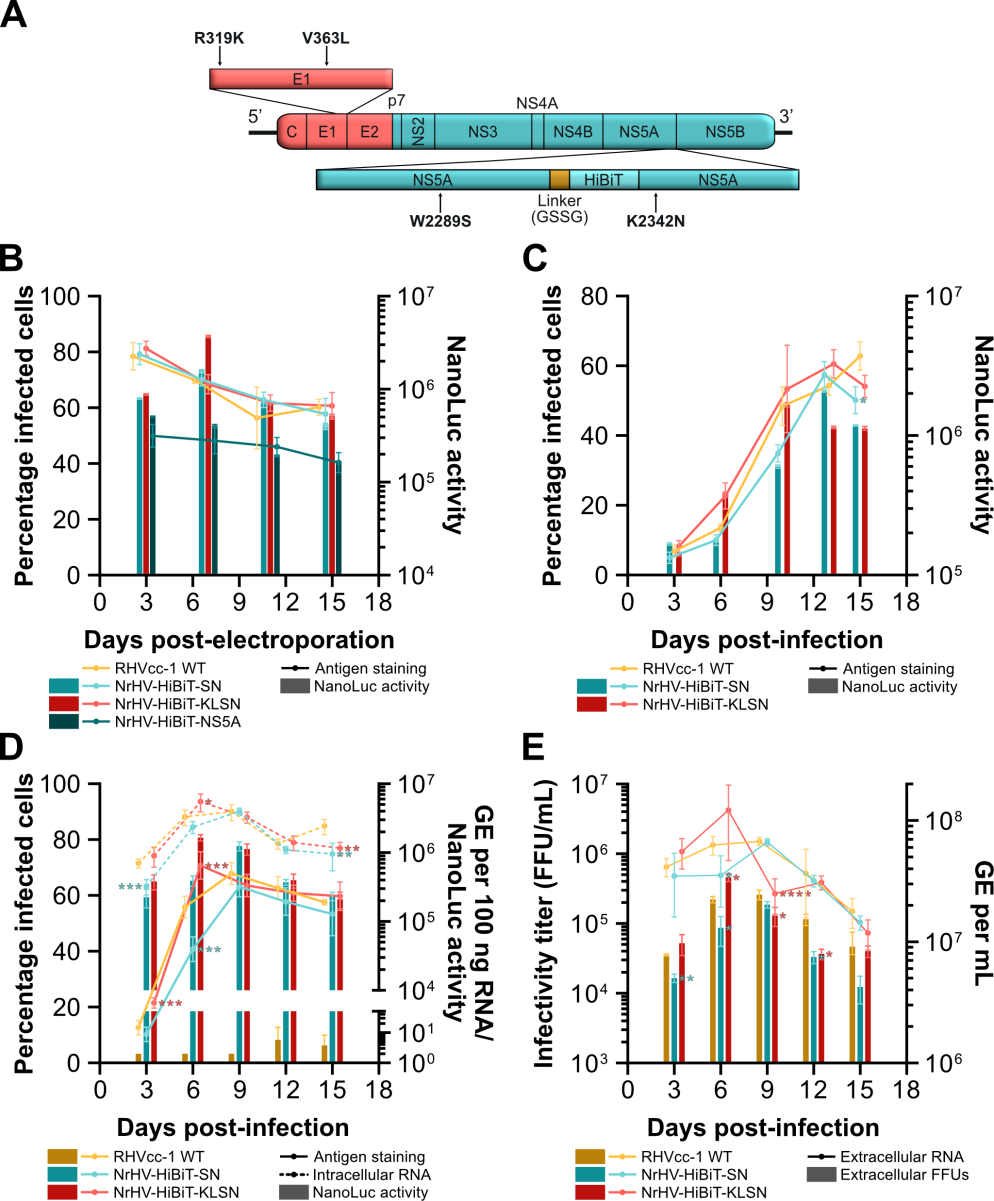
Adaptive mutations in NS5A promote fitness and stability of NrHV-HiBiT-NS5A *in vitro*. (**A**) Schematic representation of the location of the four adaptive mutations acquired by NrHV-HiBiT-NS5A. AA numbering for the labeled mutations corresponds to the RHV-rn1 polyprotein. (**B**) Electroporation of IVT RNA of RHVcc-1, NrHV-HiBiT-NS5A, NrHV-HiBiT-SN, and NrHV-HiBiT-KLSN, into McA-RH7777.hi rat hepatoma cells. Solid lines indicate manually enumerated percentage of infected cells, while bars represent NanoLuc activity subtracted mean signal from RHVcc-1-infected cells. Data points represent means ± SD from triplicates. (**C**) Infection of naïve McA-RH7777.hi rat hepatoma cells at an MOI of 0.01 with supernatant collected from cells electroporated with RHVcc-1, NrHV-HiBiT-SN, or NrHV-HiBiT-KLSN IVT RNA in the previous experiment (**B**), 11 days post-electroporation. Data points represent means ± SD from triplicates. (**D-E**) MOI infection as in (**C**) with the additional measurement of intracellular RNA titers (**D**), infectivity titers, and extracellular RNA titers (**E**). In (**D**), solid lines indicate manually enumerated percentage of infected cells, dashed lines represent intracellular RNA titers, and bars represent NanoLuc activity subtracted mean signal from reagent-only control wells. In (**E**), solid lines indicate extracellular RNA titers and bars represent viral infectivity titers calculated through manual counts of FFUs 48 h after passage onto naïve McA-RH7777.hi cells. Intracellular RNA titers represent GE per 100 ng of input RNA. Data points represent means ± SD from duplicates. Two-way ANOVA and Dunnett’s multiple comparisons test were used to determine the significance of differences of NrHV-HiBiT-SN or NrHV-HiBiT-KLSN from WT in percentage of infected cells and intracellular RNA, extracellular RNA, and infectivity titers (**C-E**). Asterisks indicate *P*-values as follows: *P ≤ 0.05, **P ≤ 0.01, ***P ≤ 0.001, ****P ≤ 0.0001. All NanoLuc activity was normalized to signal per 70,000 cells. Replicates for individual data points in (**B and C**) are technical replicates of a single electroporation or infection; those in (**D and E**) are of technical replicates of three independent infections.

To determine whether the increased percentage of infected cells and luciferase signal for NrHV-HiBiT-NS5A was due to the acquisition of adaptive mutations, we extracted RNA from supernatant collected 9 days post-infection (Fig. 2E) and sequenced the viral ORF. This uncovered four non-synonymous mutations: R319K and V363L in E1 and W2289S and K2342N in NS5A (numbering according to the RHV-rn1 polyprotein). We therefore generated NrHV-HiBiT-KLSN containing all four mutations, and NrHV-HiBiT-SN containing only the two NS5A mutations (Fig. 3A). IVT RNA of NrHV-HiBiT-SN and NrHV-HiBiT-KLSN was then electroporated into McA-RH7777.hi cells alongside the non-adapted NrHV-HiBiT-NS5A and WT RHVcc-1. NrHV-HiBiT-SN and NrHV-HiBiT-KLSN exhibited comparable percentages of antigen positive cells to WT RHVcc-1, while the non-adapted NrHV-HiBiT-NS5A was attenuated as previously observed (Fig. 3B). After passage of supernatant from electroporated cells (11 days post-electroporation) with equal MOI of 0.01 to naïve McA-RH7777.hi cells, NrHV-HiBiT-SN and NrHV-HiBiT-KLSN exhibited similar infection kinetics to WT, suggesting that the two mutations in NS5A alone adapted NrHV-HiBiT-NS5A to cell culture (Fig. 3C). To further characterize the potential impact of the HiBiT insertion and associated mutations, this kinetic experiment was repeated to also measure intracellular RNA titers and luciferase signal as a proxy for replication and extracellular RNA and infectivity titers as a proxy for virus production. The replication kinetics of NrHV-HiBiT-KLSN correlated with RHVcc-1, displaying comparable intracellular RNA titers and antigen staining that followed NanoLuc luciferase signal closely, while NrHV-HiBiT-SN replication exhibited a slight delay (Fig. 3D). NrHV-HiBiT-KLSN and RHVcc-1 additionally exhibited similar extracellular RNA titers and infectious particle production, whereas NrHV-HiBiT-SN production remained slightly delayed (Fig. 3E). Together, these results suggested that the NS5A SN mutations rescued replication of the HiBiT reporter to near WT levels. However, the two additional mutations in E1 further granted slightly increased replication and infectious virus production. Despite its slightly delayed kinetics, NrHV-HiBiT-SN was used for all further studies to avoid the presence of additional mutations in the envelope proteins, which may impact studies of entry and neutralization. To verify stability, we electroporated NrHV-HiBiT-SN RNA into McA-RH7777.hi cells and serially passaged supernatant onto naïve cells four times, each after 6–7 days. Sanger sequencing of the viral ORF identified no coding changes to the consensus sequence after 1–3 passages, and a single mutation, Q342P in E1, after four passages.

### Viability of NrHV-HiBiT recombinants *in vivo*

**Fig 4:**
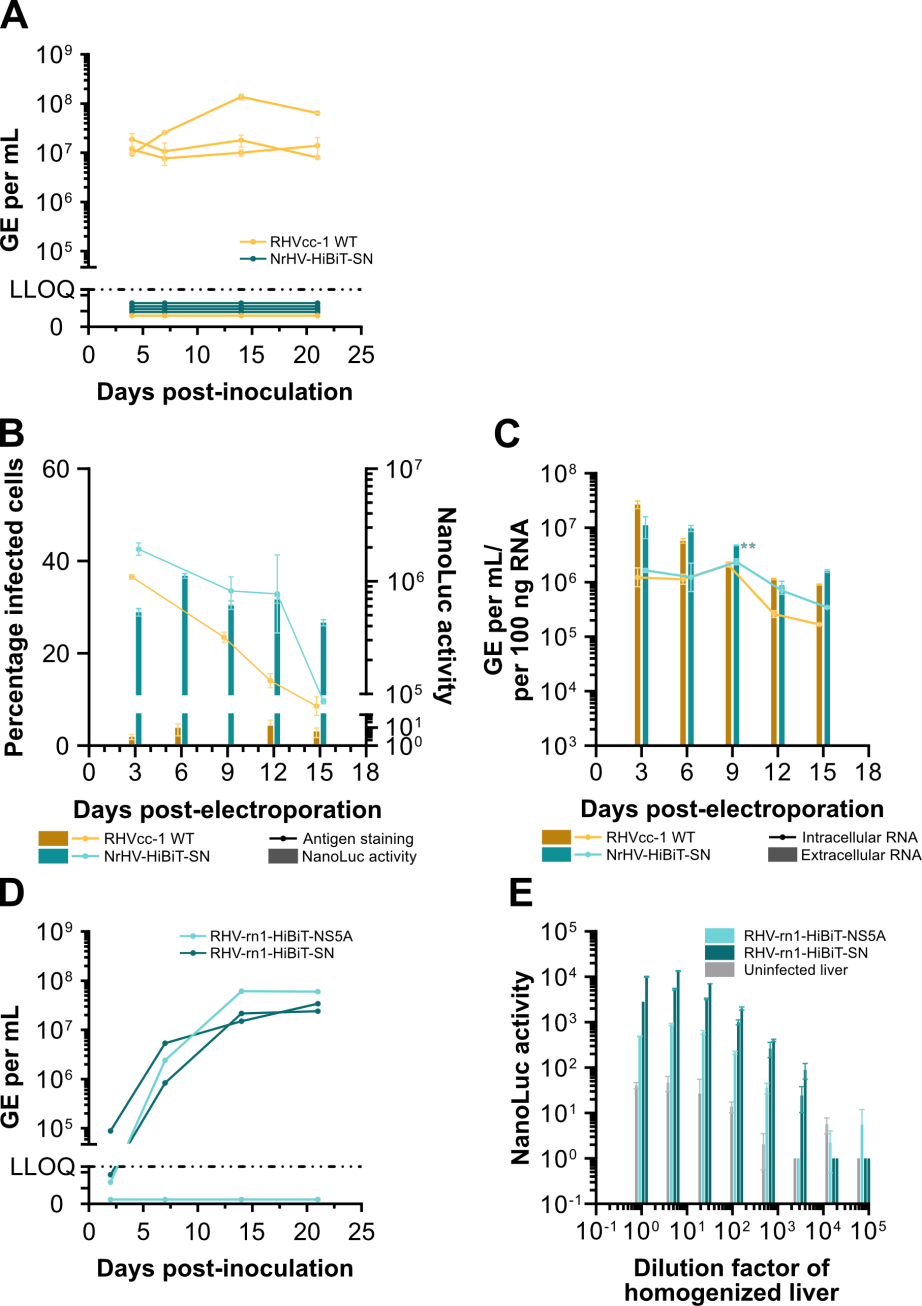
*In vivo* infection with NrHV HiBiT reporters. (**A**) Viral RNA titers in serum from CB17-SCID mice infected with 1 × 10^5^ GE of cell culture-derived RHVcc-1 or NrHV-HiBiT-SN. Data points represent means ± SD from duplicates. (**B and C**) Co-electroporation of IVT RNA of RHVcc-1 or NrHV-HiBiT-SN with miR-122 mimic into AML12 mouse hepatoma cells. In (**B**), solid lines indicate manually enumerated percentage of infected cells, and bars indicate NanoLuc activity subtracted mean signal from reagent-only control wells. In (**C**), solid lines indicate intracellular RNA titers, and bars represent extracellular RNA titers. Intracellular RNA titers represent GE per 100 ng of input RNA. NanoLuc activity was normalized to signal per 70,000 cells. Data points represent means ± SD from duplicates. Two-way ANOVA and Šídák’s multiple comparisons test were used to determine the significance of differences of NrHV-HiBiT-SN from WT RHVcc-1 in percentage of infected cells and intracellular RNA titers, and extracellular RNA titers (**B and C**). Asterisks indicate *P*-values as follows: *P ≤ 0.05, **P ≤ 0.01, ***P ≤ 0.001, ****P ≤ 0.0001. (**D**) Viral RNA titers in serum after intrahepatic injection of IVT RNA of RHV-rn1-HiBiT-NS5A or RHV-rn1-HiBiT-SN into Lewis rats. Data points represent means from technical duplicates. (**E**) NanoLuc luciferase activity subtracted mean signal from reagent-only control wells after serial dilution of liver homogenates from Lewis rats taken on day 21 from the inoculation experiment shown in (**D**). NanoLuc activity was normalized to signal per 100 mg of liver. Data points represent means ± SD from duplicates. The LLOQ for RT-qPCR was 5 × 10^4^ GE/mL. All replicates for individual data points are technical replicates for a single animal.

To permit luciferase-based readout of NrHV infection *in vivo*, we next inoculated four CB17-SCID mice each with 1 × 10^5^ genome equivalents (GE) of culture-derived NrHV-HiBiT-SN or WT RHVcc-1. While titers of ~10^7^–10^8^ GE/mL were detected for three of four WT RHVcc-1-inoculated mice throughout the course of the 21-day infection, NrHV RNA was not detectable in the sera of animals inoculated with NrHV-HiBiT-SN (Fig. 4A). To rule out a mouse-specific incompatibility caused by the HiBiT insertion, we co-electroporated IVT RNA of NrHV-HiBiT-SN or WT RHVcc-1 with miR-122 mimic into AML12 mouse hepatoma cells, a murine cell line supportive of NrHV replication upon supplementation with exogenous miR-122 (12, 15). Electroporations were followed by antigen staining and measurements of intracellular luciferase signal and RNA titers as well as extracellular RNA titers. No attenuation was seen for NrHV-HiBiT-SN compared to RHVcc-1 (Fig. 4B and C). AML12 cells are not supportive of infectious virus production, likely due to a lack of apolipoprotein E (apoE) production, and this could therefore not be assessed (12, 15). While an incompatibility of the HiBiT tag with efficient particle production in mouse cells could therefore not be excluded, these data suggested that the lack of infection in SCID mice was not due to issues with replication of the HiBiT reporter virus in mouse cells.

Given that culture-adaptive mutations do attenuate NrHV infection *in vivo* (11), we engineered the NS5A HiBiT tag into the original rat infectious RHV-rn1 clone, with or without the SN mutations (RHV-rn1-HiBiT-SN and RHV-rn1-HiBiT-NS5A). Viral RNA from these constructs was intrahepatically injected into two Lewis rats per construct, and viral titers were followed for 3 weeks. Both animals inoculated with RHV-rn1-HiBiT-SN and one animal inoculated with RHV-rn1-HiBiT-NS5A had detectable titers by 7 days post-inoculation. NrHV RNA titers increased to >1 × 10^7^ GE/mL in all three animals 14 days post-inoculation (Fig. 4D). Sanger sequencing of the HiBiT insertion region confirmed that no changes were observed over the course of infection. Furthermore, NanoLuc luciferase signal was detected from the homogenized livers of all NrHV-positive rats (Fig. 4E). The NanoLuc luciferase signal from the livers of both animals infected with RHV-rn1-HiBiT-SN was >10-fold higher than that of the animal infected with RHV-rn1-HiBiT-NS5A, suggesting the two mutations in NS5A also improved expression of the HiBiT tag *in vivo*.

### The NrHV HiBiT reporter allows high-throughput, accurate quantification of neutralizing antibodies

**Fig 5:**
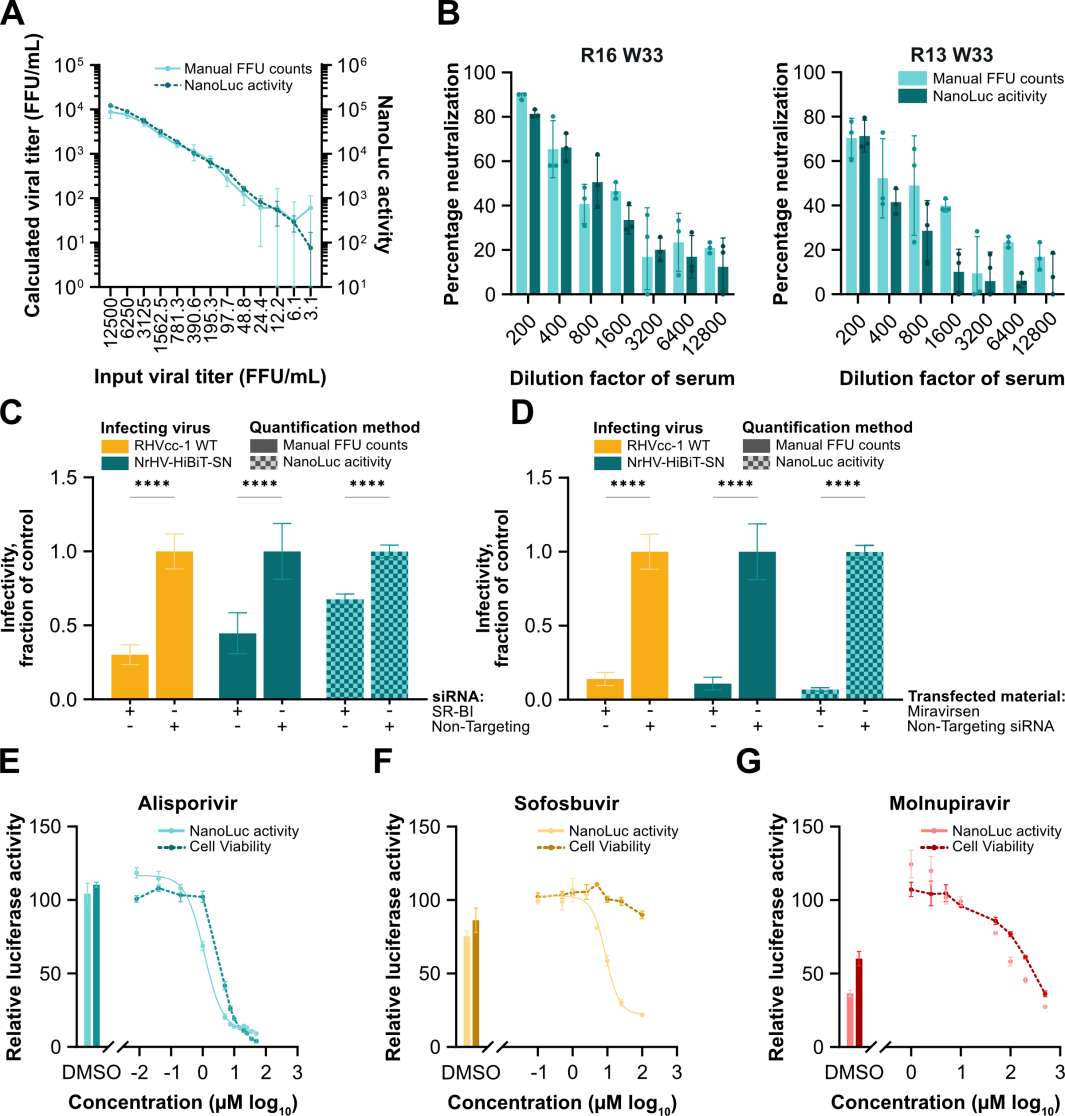
High-throughput quantification of neutralizing antibodies, host factor inhibitors, and antiviral compounds using the NrHV-HiBiT-SN reporter. (**A**) Infection of McA-RH7777.hi rat hepatoma cells with decreasing titers of NrHV-HiBiT-SN. Viral infectivity titers were calculated through manual counts of FFUs after 48 h and compared to NanoLuc luciferase signal. Data points represent means ± SD from triplicates. (**B**) NrHV-HiBiT-SN was exposed to dilutions of sera from chronically infected rats (R16 week 33, R13 week 33, [11]) before infection of McA-RH7777.hi cells. Percentage neutralization compared to incubation with only culture medium was quantified through either manual counts of FFUs or NanoLuc luciferase. Data points represent means ± SD from triplicates. (**C and D**) Assessment of viral infection after transfection of siRNA or LNA to knock down SR-BI (**C**) or inhibit miR-122 (**D**) relative to that from cells transfected with non-targeting siRNA. Bars of solid color indicate manually enumerated FFUs, while checkered bars represent NanoLuc activity. Data points represent means ± SD from eight replicates. (**E–G**) NanoLuc luciferase signal from McA-RH7777.hi cells electroporated with NrHV-HiBiT-SN treated with increasing doses of alisporivir (**E**), sofosbuvir (**F**), or molnupiravir (**G**). Values are given as percentage luciferase signal relative to untreated control wells. DMSO-only controls are shown for comparison. In addition, luciferase measurements of cell viability are given relative to untreated control wells using the CellTiter-Glo luminescent cell viability assay. Data points represent means ± SD from triplicates. Two-way ANOVA and Šídák’s multiple comparisons test were used to determine the effects of siRNA or LNA transfection (**C-D**). Asterisks indicate *P*-values as follows: *P ≤ 0.05, **P ≤ 0.01, ***P ≤ 0.001, ****P ≤ 0.0001. All replicates for individual data points are technical replicates of a single electroporation or infection.

To establish the correlation between NanoLuc signal and viral infectivity titers, McA-RH7777.hi cells were infected with increasing doses of NrHV-HiBiT-SN. Infection was quantified by manual counting of FFUs or by luciferase signal. The quantification correlated closely between the two methods across the dilution series, with luciferase exhibiting lower levels of variation at the highest dilutions (Fig. 5A). To validate the utility of the NrHV-HiBiT-SN reporter for efficient quantification of nAbs, we used the previously described *in vitro* neutralization assay (11, 17) and compared quantification by manual FFU counting and luciferase signal. Quantification of nAbs in serum from persistently infected rats was in high agreement between NanoLuc luciferase and manual FFU counts across the dilution series for the sera tested. Less variation was observed from quantification by luciferase (Fig. 5B). Accordingly, NrHV-HiBiT-SN presents a high-throughput platform for the quantification of neutralizing antibodies.

### Efficient assessment of host factor interactions and antiviral compounds using the NrHV HiBiT reporter

To also demonstrate the utility of the HiBiT reporter in studies of interactions with host factors, we next focused on the entry factor SR-BI and the replication factor miR-122. McA-RH7777.hi cells were transfected with either SR-BI-targeting siRNA, non-targeting siRNA, or the miR-122 inhibitor miravirsen, prior to infection with WT RHVcc-1 or NrHV-HiBiT-SN. Quantification of NrHV-HiBiT-SN infection using luciferase again correlated well with the manual FFU counts of both WT RHVcc-1 and NrHV-HiBiT-SN infections for SR-BI siRNA and miravirsen-treated cells (Fig. 5C and D). To next assess whether cyclophilin A (CypA), a cis-trans isomerase and host factor for HCV (28), also plays a role for NrHV, we electroporated McA-RH7777.hi cells with NrHV-HiBiT-SN prior to exposure to the CypA inhibitor, alisporivir (29). Although alisporivir had a clear dose-dependent effect on NrHV infection with an EC_50_ of 1.12 µM, the therapeutic window was narrow with apparent cytotoxicity at <10-fold higher doses (Fig. 5E). Nonetheless, this suggested that CypA may act as a host factor for NrHV, similarly to HCV.

To similarly establish the utility of NrHV-HiBiT-SN to quantify the activity of antiviral compounds, NrHV-HiBiT-SN was electroporated into McA-RH7777.hi cells and exposed to the HCV nucleotide/nucleoside inhibitors, sofosbuvir or molnupiravir (30, 31). A dose-dependent antiviral effect, without cytotoxicity, was observed for sofosbuvir against NrHV-HiBiT-SN (Fig. 5F). Luciferase-derived results yielded an EC_50_ of 8.92 µM, correlating with the previously determined EC_50_ of 16 µM obtained for an NrHV sub-genomic replicon (NrHV-SGR) (9). Molnupiravir did not exhibit antiviral effects at non-cytotoxic concentrations (Fig. 5G). These findings highlight the utility of the NrHV-HiBiT-SN reporter as a tool for use in the characterization of virus-host interactions and antiviral compounds for this hepacivirus model.

## DISCUSSION

In this study, we generated an NrHV reporter virus for use in high-throughput analyses. While the insertion of full-length reporter genes proved to be highly attenuating, the insertion of the HiBiT tag (18) into NS5A domain III of the culture-adapted RHVcc-1 (11) produced a reporter virus (NrHV-HiBiT-SN) with near WT viability *in vitro* after the acquisition of two mutations in NS5A. Engineering the HiBiT tag into NS5A of the original *in vivo* infectious RHV-rn1 clone (10, 13) further permitted productive infection in rats. The NrHV-HiBiT-SN reporter facilitated straightforward quantification, providing means to study viral replication, neutralizing antibodies, and host factor interactions in a high-throughput manner. Using this reporter, we identified CypA as a potential novel host factor for NrHV. These findings expand our knowledge of NrHV as an HCV model and demonstrate the utility of this reporter in the further characterization and study of NrHV.

Initial attempts to insert full-length FLuc or EGFP into culture-adapted NrHV revealed key differences in the tolerance of insertions into the viral genome between HCV and NrHV. Despite each of the four insertion sites explored in this study having been inspired by functional HCV reporters (20–22), insertions of reporter genes at none of these sites were tolerated for NrHV replication. A transposon screen, as done for HCV and other viruses (32), could potentially aid in the discovery of acceptable insertion sites for NrHV and clarify the distinct differences between NrHV and HCV with regards to the tolerance of genomic modifications.

For HCV, the initial nucleotides of the ORF constitute part of domain IV of the IRES and are critical for efficient translation (33). While a similar structure has not been predicted for NrHV (13), this could explain why both HiBiT-Core reporters were replication incompetent, whereas an NrHV-SGR with FLuc insertions following the first 12 codons replicated efficiently (9). While the HiBiT insertions may have also altered the integrity and function of the core protein itself, that would not be predicted to impact RNA replication. Experimental characterization of the secondary structures of NrHV RNA would be needed to understand the role of the first nucleotides of the ORF in viral replication and translation.

The insertion of the HiBiT tag at the N-terminus of NS2 did not affect replication, consistent with observations that NS2 itself is not critical for HCV replication (34). It did, however, disrupt virus production, which is in accordance with the importance of NS2 for HCV virion morphogenesis and assembly (21, 35), with mutations in the first 5 AA residues heavily attenuating infectious virus production (36). This N-terminal insertion may therefore interfere with homologous functions for NrHV. The insertion of the HiBiT tag at this site was, however, permitted for HCV (24), alluding further to discrepancies in the tolerance to ORF modifications between the two viruses.

The NrHV-HiBiT-NS5A reporter was capable of both replication and infectious particle production *in vitro*. The insertion site in NS5A domain III was inspired by the insertion of GFP at the corresponding site in HCV NS5A (25, 26) and selected after alignment of NrHV NS5A with that of several HCV genotypes. The dissimilarity of NrHV and HCV in this region complicated the identification of corresponding sites, and the NrHV insertion may therefore not correspond exactly to that in HCV. Nonetheless, the large variability in this region, even among HCV genotypes (37), may suggest plasticity and flexibility for insertions. Of the four identified putative adaptive mutations for the NrHV-HiBiT-NS5A reporter, only the two mutations in NS5A were necessary to accommodate the HiBiT tag, with NrHV-HiBiT-SN exhibiting near WT kinetics of replication and particle production *in vitro*. The presence of the two NS5A mutations led to increased replication and luciferase production immediately following RNA electroporation (Fig. 3B). We further speculate that their proximity to the HiBiT insert could indicate a direct compensatory role for changes in protein conformation or interactions affected by the HiBiT insert, and that they, due to their location in NS5A domain III, further may impact infectious virus production (37, 38). The additional two E1 mutations led to accelerated kinetics after infection, including increased infectious particle production (Fig. 3C through E). These mutations have not been observed in previous studies, although V363L in E1 is in close proximity to several mutations involved in the adaptation of NrHV to mice (10). NrHV-HiBiT-SN proved stable *in vitro*, acquiring only the single Q342P mutation in E1 after four passages; this culture-adaptive mutation was previously observed for NrHV-B and NrHV-K (15, 16). This suggested an only weak pressure for further adaptation of NrHV-HiBiT-SN, and that mutations other than the KL mutations could accommodate this. In the interest of avoiding changes to the envelope proteins, we did not pursue further passaging but note that such may lead to accumulation of fitness-enhancing mutations, particularly in the envelope genes, and possibly also loss of the reporter gene (26). However, the short nature of the HiBiT tag likely reduces this risk significantly.

The lack of infection in SCID mice with culture-derived NrHV-HiBiT-SN was unexpected, given successful infections with RHVcc-1 (11). Upon electroporation of AML12 mouse hepatocytes, NrHV-HiBiT-SN and RHVcc-1 demonstrated similar replication (Fig. 4B and C). Also given their identical envelope genes, this did not suggest that mouse-specific attenuation of the reporter caused the inability to infect or replicate in SCID mice. However, since AML12 cells do not permit infectious particle production (12, 15), likely due at least in part to lack of apoE expression (12, 39), a mouse-specific defect of NrHV-HiBiT-SN particle production cannot be excluded. Nonetheless, we speculate that the combination of cell culture adaptive mutations and the insertion of the HiBiT tag may have attenuated the reporter virus beyond recovery. Correspondingly, when re-engineered into the RHV-rn1 rat infectious clone (13), intrahepatic inoculation of both RHV-rn1-HiBiT-NS5A and RHV-rn1-HiBiT-SN RNA led to productive infection in rats. Whereas RHV-rn1 infection or intrahepatic RNA inoculation typically leads to serum viremia of ~10^9^ GE/mL in rats (11, 17), levels for the HiBiT reporters only reached 10^7^–10^8^ GE/mL. Long-term infection in rats may reveal further *in vivo* adaptive mutations to increase fitness. Nonetheless, the HiBiT sequence remained unchanged in both reporters over the 3-week course of infection; stability over the entirety of the ORF was not assessed. Additionally, luciferase signal was observed from infected rat livers in a dose-dependent manner, confirming the integrity of the HiBiT tag. While RHV-rn1-HiBiT-NS5A infection resulted in slightly higher serum viremia compared to RHV-rn1-HiBiT-SN, we observed ~10-fold higher luciferase signal from the livers of animals infected with RHV-rn1-HiBiT-SN. Although based on few animals, this may suggest that the SN mutations also compensate for the insertion of the HiBiT tag by leading to increased replication *in vivo*, perhaps at the expense of virus production. Transmission of infectious virus between co-housed animals has so far not been observed (11, 17) and was therefore not expected to influence these observations. While this demonstrated the viability of the NrHV-HiBiT-SN reporter *in vivo*, direct luciferase monitoring would depend on co-expression of the complementary LgBiT protein, for example, in a transgenic mouse model. Although the HiBiT system has been utilized for live imaging of viral infection *in vivo*, permitted by the prior injection of LgBiT-expressing tumor cells (40), the delivery of the LgBiT protein itself to the tissue of interest has proven difficult (41). Future development of a virus containing a full-length reporter gene would therefore still be of interest for the utilization of live imaging *in vivo*.

NrHV infection was reliably and more stably measured using the NrHV-HiBiT-SN reporter compared to manual FFU counts. Accordingly, the NrHV reporter provides a high-throughput method for the assessment of the neutralizing capability of antibodies, a tool that can accelerate ongoing vaccine studies using the NrHV model. More broadly, we demonstrate the benefit of this reporter in the study of viral host factors, with comparable outcomes from luciferase signal and manual FFU counts during knock-down or depletion of the NrHV host factors SR-BI and miR-122 (9–11). The lack of additional envelope mutations avoids further issues in interpreting data, for example, from studies of neutralization or additional HCV entry factor orthologs such as CD81, claudins, and OCLN (12). The automated process of luciferase measurement further reduces the influence of human error and bias on data collection. CypA is a critical host factor for HCV, providing a target for host-targeting therapeutic approaches (42). While the current data indicated that CypA may similarly be a host factor for NrHV, the narrow dose window observed for the antiviral effects of alisporivir in the absence of cytotoxicity prevents a firm conclusion. The sensitivity of infections to alisporivir also differed, with NrHV-HiBiT-SN having an EC_50_ of 1.12 µM compared to 0.03 µM for HCV (43). Although this could be indicative of divergent reliance on CypA, it could also be the effect of lower efficacy of alisporivir-mediated inhibition of rat compared to human CypA or reflect differences in cellular uptake. This finding nonetheless corroborates the use of this reporter in the study of virus-host interactions. Finally, luciferase measurements revealed an EC_50_ of 8.92 µM for the inhibition of NrHV by sofosbuvir, corresponding to previous observations from the NrHV sub-genomic replicon (9). For molnupiravir, we did not observe antiviral effects at non-cytotoxic concentrations.

In summary, we generated a stable NS5A-HiBiT-tagged NrHV reporter virus permitting high-throughput studies of viral infection, yielding results in concordance with previous manual methods. This work thereby provides a flexible high-throughput tool for the NrHV model, facilitating the study of host factors, antivirals, and nAbs. NrHV holds great promise as a model for HCV, permitting the study of intrahepatic pathology in an immunocompetent setting as well as the assessment of vaccine platforms and candidates in a rodent challenge model. Future work to similarly introduce the HiBiT tag into other NrHV strains (16) would further allow high-throughput assessment of nAb cross-reactivity, furthering work toward a multivalent vaccine. Knowledge from the NrHV model on the best performing vaccine candidates, or combinations thereof, could then be applied with greater confidence to trials in high-incidence HCV patient cohorts (44).

## MATERIALS AND METHODS

### Animal experiments

For infection experiments, female CB17-SCID (CB-17/Icr-Prkdcscid/scid/Rj) mice were inoculated intraperitoneally with 1 × 10^5^ GE of cell culture-derived NrHV under isoflurane anesthesia. RNA launch in rats was conducted in female LEWOrl/Rj rats (Janvier) by percutaneous intrahepatic injection under isoflurane anesthesia of 10 µg IVT NrHV genomic RNA dissolved in 100 µL sterile phosphate-buffered saline (PBS) distributed in two hepatic locations, each receiving 5 µg. Mice were bled via facial vein puncture, while rat blood was sampled by tail vein puncture under isoflurane anesthesia according to guidelines for blood sampling. All animals had access to food (SAFE D03, SAFE Complete Care Competence, Rosenheim, Germany) and water *ad libitum* and were housed by gender (except for breeders) in Innovive IVC caging containing wood chip bedding, shelters, nesting material, and biting sticks on a 12-h light-dark cycle. All experimentation was conducted during the light cycle.

### Cells and antibodies

McA-RH7777.hi rat hepatoma cells permissive to infection by NrHV (9) were maintained in DMEM (Thermo Fisher Scientific) supplemented with 10% FBS, 100 U/mL penicillin, and 100 µg/mL streptomycin (Pen Strep: Sigma-Aldrich) at 37°C, 5% CO_2_. AML12 cells were maintained in DMEM-F12 (Thermo Fisher Scientific) supplemented with 10% FBS, 100 U/mL penicillin, 100 µg/mL streptomycin (Pen Strep: Sigma-Aldrich), 1× insulin-transferrin-selenium (ITS-G, Thermo Fisher Scientific), and 40 ng/mL dexamethasone (Thermo Fisher Scientific). Cells were passaged every 2–3 days using trypsin-EDTA (Sigma-Aldrich).

Polyclonal NrHV antibody was obtained by purification of total IgG from sera collected from C57BL/6 mice 6 weeks post-infection, following viral clearance after inoculation with mouse-adapted NrHV (15).

### Cloning of NrHV constructs

All NrHV reporter constructs were generated through the ligation of single or multiple amplicons using the In-Fusion Snap Assembly Master Mix (Takara Bio), following the manufacturer’s instructions, or through megaprimer mutagenesis unless otherwise stated. All megaprimer fragments were gel-purified using the Zymoclean Gel DNA Recovery Kit (Zymo Research). Megaprimer PCR amplifications were completed using 200 ng of purified megaprimer fragment and 50 ng of template plasmid with consistent thermocycler conditions (1 cycle of 98°C for 30 s; 20 cycles of 98°C for 10 s, 48°C for 1 min, 72°C for 20 min; 1 cycle of 72°C for 20 min; hold at 4°C). All PCRs were carried out using Q5 Hot Start High-Fidelity 2X Master Mix (New England Biolabs) unless using a QuikChange Site-Directed Mutagenesis Kit (Agilent Technologies), for which the proprietary polymerase was used. All clones were sequence verified over the entirety of the viral genome using Sanger sequencing (Macrogen).

To engineer an RHVcc-1 reporter with CpG/UpA-optimized FLuc (19) inserted at the N-terminus of core, primer pair TS-O-01253/TS-O-01254 (Table S1) was used to amplify the 5′ UTR and FLuc insert from a monocistronic NrHV-SGR (9), including a GSG linker and P2A sequence as a 3′ overhang. The remaining sequence of the cell culture-adapted RHV-rn1 plasmid, pRHVcc-1 (11), was amplified with the primer pair TS-O-02155/TS-O-01256, and the two fragments were ligated. The resulting plasmid contained a frameshift mutation that was corrected using a megaprimer generated from primer pair TS-O-01283/TS-O-00260. To insert *Rattus norvegicus* ubiquitin downstream of the P2A sequence, this frameshift-corrected reporter was linearized using the primer pair TS-O-01425/TS-O-01426 and ligated with *Rattus norvegicus* ubiquitin synthesized by IDT (IDT_Ubi_BuildingBlock, Table S1).

Insertion of CpG/UpA optimized FLuc or EGFP between p7 and NS2 was achieved through ligation of linearized pRHVcc-1, amplified using the primer pair TS-O-01221/TS-O-01222, and CpG/UpA optimized FLuc or EGFP amplified from NrHV sub-genomic replicons containing the appropriate reporter gene (9) using primer pairs TS-O-01223/TS-O-01224 or TS-O-01227/TS-O-01228.

To insert CpG/UpA optimized FLuc in between duplicated NS5A/NS5B cleavage sites, the sequence and plasmid backbone of an RHV-rn1 construct containing the S1757A and T2373A cell culture-adaptive mutations (11) and the FLuc reporter gene from a RHV-rn1-SGR construct (9), containing a fragment of the NS5A/NS5B cleavage site at the 5′ and 3′ ends, were amplified using the primer pairs TS-O-00889/TS-O-00890 and TS-O-01098/TS-O-01099 respectively, and were subsequently ligated. CpG/UpA-optimized EGFP was inserted at the same site using an 800 bp megaprimer amplified from an RHV-rn1-SGR-EGFP construct (9) using the primer pair TS-O-00305/TS-O-00306, similarly containing the NS5A/NS5B cleavage site at both 5′ and 3′ ends. Missing cell culture adaptive mutations were introduced to both reporter constructs using a 5387 bp megaprimer including the L586S mutation, amplified from the same RHV-rn1 construct containing the S1757A and T2373A cell culture-adaptive mutations (11) using primer pair TS-O-01105/TS-O-01106. The T2373A mutation was engineered into the 2nd NS5A/NS5B cleavage site using the QuikChange Lightning Site-Directed Mutagenesis Kit (Agilent Technologies) following the manufacturer’s instructions with the primer pair TS-O-01184/TS-O-01185, thereby placing this adaptive mutation in both repeats of the cleavage site.

To insert the HiBiT tag into RHVcc-1, we ligated amplicons of the entirety of the RHVcc-1 genome and plasmid backbone amplified by the primer pairs TS-O-01591/TS-O-01592, TS-O-01597/TS-O-01598, and TS-O-01599/TS-O-01600 containing the HiBiT tag and linker as overhangs at the 5′ and 3′ ends. Primer pair TS-O-01595/TS-O-01596 enabled amplification of *Rattus norvegicus* ubiquitin; thus, ligation with the full-length RHVcc-1 amplicon produced by the primer pair TS-O-01593/TS-O-01594 permitted the insertion of ubiquitin downstream of the HiBiT tag and GSSG linker.

To generate the NrHV-HiBiT-SN and NrHV-HiBiT-KLSN mutants of NrHV-HiBiT-NS5A, the primer pairs TS-O-01713/TS-O-01714 and TS-O-01711/TS-O-01712 were used to create amplicons containing the desired mutations within E1 and NS5A, respectively. These amplicons were used for megaprimer amplification, allowing mutation of the first two positions in NS5A and subsequently the two additional positions in E1.

The RHV-rn1-HiBiT-NS5A clone was produced through ligation of an amplicon of the entirety of the RHV-rn1 plasmid produced by the primer pair TS-O-01599/TS-O-01600, introducing the HiBiT tag and GSSG linker to NS5A domain III.

To produce the RHV-rn1-HiBiT-SN mutant, HiBiT-adaptive mutations were introduced to RHV-rn1-HiBiT-NS5A using two methods. The W2289S mutation in NS5A was engineered using the megaprimer method described above, and the K2342N mutation was added using the QuikChange XL Site-Directed Mutagenesis Kit (Agilent Technologies) following manufacturer’s instructions with the primer pair TS-O-02128/TS-O-02129.

### RNA *in vitro* transcription, transfection, and electroporation

Viral RNA transcripts for electroporation were generated from 2.5 µg of *MluI*-linearized plasmid using the T7 RiboMAX express large-scale RNA production system (Promega), before being DNase treated with RQ1 RNase-free DNase (Promega) for 30 min on ice. IVT-generated RNA was then purified using RNA Clean & Concentrator-25 (Zymo Research) prior to storage at −70°C.

Prior to transfections, 300,000 McA-RH7777.hi cells (9) were seeded in six-well plates pre-coated with 0.1% gelatin, prepared from porcine gelatin powder (Sigma-Aldrich) dissolved in MilliQ water. To transfect IVT RNA, Lipofectamine 2000 transfection reagent (Thermo Fisher Scientific) was diluted in Opti-MEM (Thermo Fisher Scientific) at a ratio of 1:50. For each construct to be transfected, 5 µg IVT RNA was then diluted in 250 µL Opti-MEM. Diluted Lipofectamine 2000 transfection reagent and IVT RNA were then mixed at a ratio of 1:1 and incubated at room temperature for 20 min. Growth media was then removed from wells containing the previously seeded McA-RH7777.hi cells. Cells were washed with PBS and 1.5 mL Opti-MEM was added before 500 µL of the transfection mixture was gently pipetted onto cells. Plates were then incubated at 37°C, 5% CO_2_.

For electroporation, McA-RH7777.hi cells (9) or AML12 cells were washed twice with ice-cold PBS before being resuspended at a cell density of 1.5 × 10^7^ cells/mL in either PBS supplemented with 1:50 ATP and 1:50 glutathione or cytomix (120 mM KC1; 0.15 mM CaCl_2_; 10 mM K_2_HPO_4_/KH_2_PO_4_, pH 7.6; 25 mM HEPES, pH 7.6; 2 mM EGTA, pH 7.6; 5mM MgCl_2_, pH adjusted with KOH; 2mM ATP and 5mM glutathione). Once diluted, 400 µL of cell suspension was added to 5 µg of IVT RNA and transferred to a 4 mm electroporation cuvette (Bio-Rad). For electroporation of AML12 cells, cell suspensions were added to 5 µg of IVT RNA and 0.325 µM miR-122 mimic (miRIDIAN mimics, Horizon Discovery LTD). The following parameters on a Bio-Rad Gene Pulser XCell electroporation system were used for exponential decay electroporation: 270 V, 975 µF, ∞ ohm. Electroporated cells were immediately resuspended in the appropriate media pre-warmed to 37°C and subsequently transferred to 15 cm culture dishes coated with filtered 0.1% gelatin. Post-electroporation, cells were split and supplied with fresh media every 2 days.

To maintain NrHV replication in AML12 cells, an additional miR-122 mimic was delivered by reverse transfection every 3 days. Lipofectamine RNAiMAX transfection reagent (Thermo Fisher Scientific) was diluted in Opti-MEM (Thermo Fisher Scientific) at a ratio of 1:16.67. miR-122 mimic was diluted in Opti-MEM to achieve a final concentration of 0.325 µM. Dilutions of RNAiMAX and miR-122 mimic were mixed at a ratio of 1:1 and incubated at room temperature for 10 min. The transfection mixture was then added to a suspension of previously electroporated AML12 cells that were then seeded at the desired concentration onto 12-well plates pre-coated with 0.1% gelatin.

### Transfection of siRNA and LNA

To transfect McA-RH7777.hi cells (9) with siRNA or miravirsen (45), 300,000 cells were seeded in six-well plates pre-coated with 0.1% gelatin. Lipofectamine RNAiMAX transfection reagent (Thermo Fisher Scientific) was then diluted in Opti-MEM (Thermo Fisher Scientific) at a ratio of 1:16.67. SR-BI-targeting siRNA (ON-TARGETplus SMARTpool, Horizon Discovery LTD), non-targeting siRNA pool (ON-TARGETplus Non-targeting siRNA #4), or miravirsen (+CC*+AT*T*+G + TC*A*+CA*+CT*+C + C; + designating LNA bases and * phosphorothioate DNA backbone; Qiagen) were reconstituted in RNase-free water and diluted in Opti-MEM to the desired concentration for transfection. Diluted siRNAs and LNAs were mixed with diluted Lipofectamine RNAiMAX transfection reagent at a ratio of 1:1 and left to incubate at room temperature for 5 min. Onto each well containing seeded cells covered in 1.7 mL DMEM supplemented with 3% FBS, 100 U/mL penicillin, and 100 µg/mL streptomycin (Pen Strep: Sigma-Aldrich), 300 µL of this transfection mixture was carefully pipetted prior to incubation for 4–6 h at 37°C, 5% CO_2_. Transfection of siRNA and LNA was carried out twice, 3 days and 1 day prior to infection with NrHV constructs.

### Extraction, quantification, and sequencing of viral RNA

To extract viral RNA from supernatant of infected cell cultures, 250 µL of supernatant was mixed with 750 µL TRIzol LS reagent (Thermo Fisher Scientific) and incubated for 5 min at room temperature. For the extraction of viral RNA from rodent serum samples, 25 µL of the sera was diluted in 225 µL chilled PBS prior to mixing with TRIzol LS reagent. To this mixture, 200 µL of chloroform was added before being shaken vigorously for 15 s and left at room temperature for a further 3 min before being centrifuged at 12,000 × *g* for 15 min at 4°C. After centrifugation, the aqueous phase was recovered and mixed with 450 µL of anhydrous ethanol and transferred to an RNA Clean & Concentrator-5 column (Zymo Research), with RNA purification and concentration being carried out following the manufacturer’s instructions.

To extract RNA from pelleted cells for determination of viral intracellular RNA titers, pelleted cells were processed using the RNeasy mini kit (Qiagen) following the manufacturer’s instructions, eluting into a final volume of 30 µL.

Detection and quantification of NrHV RNA was achieved through RT-qPCR with TaqMan Fast Virus 1-Step Master Mix (Thermo Fisher Scientific) using a LightCycler 480 thermocycler running the following protocol: 50°C for 30 min, 95°C for 5 min, followed by 40 cycles of 95°C for 15 s, 56°C for 30 s, and 60°C for 45 s, and finally 40°C for 10 s. Amplification was achieved using the primers TS-O-00561 and TS-O-00562 and probe TS-O-00563 (Table S1).

For whole ORF sequencing, following RNase inhibition by incubation with RNasin Plus RNase inhibitor (Promega) for 2 min at 65°C, cDNA of the viral ORF was generated from extracted RNA using Maxima Minus H Reverse Transcriptase (Thermo Fisher Scientific) with TS-O-00319 as a 3′ cDNA primer, being incubated for 120 min at 50°C followed by 5 min at 85°C. RNA was degraded through a 20-min incubation at 37°C with RNase H and RNase T1 (Thermo Fisher Scientific). The viral ORF was amplified using Q5 Hot Start High-Fidelity DNA Polymerase (New England Biolabs) with the primers TS-O-00316 and TS-O-00318 and the following thermocycler settings: 98°C for 30 s, 37 cycles of 98°C for 10 s, 65°C for 10 s, 72°C for 8 min, and a final cycle of 72°C for 10 min before being held at 4°C. Amplified ORFs were sent for Sanger sequencing (Macrogen).

### Detection of luminescence and fluorescence

To measure FLuc activity, cells were washed once with PBS after the removal of growth media and then lysed with passive lysis buffer. Luciferase was then measured with the luciferase assay system (Promega) following the manufacturer’s instructions using a CLARIOstar luminometer (BMG Labtech).

To inspect the EGFP signal, cells were seeded in eight-chamber slides. DMEM (Thermo Fisher Scientific) was replaced with FluoroBrite DMEM (Thermo Fisher Scientific) once cells had settled to reduce autofluorescence. Chamber slides were manually inspected for fluorescent cells using a Carl Zeiss Axio Vert. A1 microscope.

For measurement of NanoLuc luciferase activity using the HiBiT Nano-Glo detection system (Promega), Nano-Glo Lytic Reagent was prepared following the manufacturer’s instructions. Where luciferase was to be detected from cells seeded in 96-well plates, opaque white 96-well cell culture plates were coated by a 2-h incubation with 50 µL of 10 µg/mL laminin. Plates were washed three times with PBS to remove any unbound laminin before seeding McA-RH7777.hi cells, with a medium volume of 50 µL. For detection of luciferase from pelleted cells, cells were first resuspended in PBS to the cell concentration at which they were pelleted, with 50 µL of the resuspension being added to an opaque white 96-well luciferase plate. An equal volume of Nano-Glo Lytic Reagent was added to the samples and mixed by pipetting. Plates were placed on an orbital shaker for at least 10 min prior to detection and quantification of luminescence using a CLARIOstar luminometer (BMG Labtech).

To measure NanoLuc luciferase signal from infected Lewis rat livers, liver sections weighing ~100 mg after trimming of fat and connective tissue were transferred to a 1.5 mL screw cap tube and mechanically homogenized in 500 µL PBS using a 1.5 mL disposable pestle. Homogenates were then centrifuged for 3 min at 100 g, 4°C, before being diluted as indicated. Dilutions were added to an opaque white 96-well luciferase plate and processed as above. Luciferase measurements were adjusted to control for variation in weight between liver samples.

### Infectivity titration

To determine infectivity titers, 13,500 McA-RH7777.hi rat hepatoma cells (9) were seeded per well into laminin-coated 96-well cell culture plates. Supernatants from infected cell cultures were thawed and diluted to create a 10-fold dilution series from 1:2 to 1:2,000. Each dilution of virus stock was applied to the cells in triplicate, and cells were incubated at 37°C, 5% CO_2_, for 48 h, after which antigen staining of NrHV was carried out with NrHV-specific mouse IgG and Alexa Fluor 594 goat-anti-mouse IgG as previously described (11, 15, 17). Images were captured at 50× magnification using a Carl Zeiss Axio Vert. A1 microscope. The FFU/mL titer was enumerated manually, with a single FFU being defined as a group of more than one infected cell with a distance of at least two uninfected cells from another FFU.

### Neutralization assay

To determine the neutralizing capacity of NrHV-specific antibodies in serum from infected animals, 13,500 McA-RH7777.hi cells (9) were seeded per well into laminin-coated 96-well cell culture plates. Serum from infected animals was heat-inactivated at 56°C for 30 min prior to use. Serum was then diluted 1:100 in DMEM supplemented with 10% FBS, 100 U/mL penicillin, and 100 µg/mL streptomycin (Pen Strep: Sigma-Aldrich), with this dilution being further serially diluted 1:2 to acquire dilutions ranging from 1:100 to 1:6,400. Virus and dilutions of sera were mixed in an empty 96-well plate at a ratio of 1:1 and incubated for 1 h at 37°C, 5% CO_2_. For positive controls, the virus was mixed with DMEM at a ratio of 1:1, and for negative controls, only DMEM was used. After 1 h, the serum/virus mix and controls were transferred onto the seeded McA-RH7777.hi cells and incubated at 37°C, 5% CO_2_ for 4 h. Cells were then washed twice with PBS, covered with 100 µL DMEM, and incubated for a further 44 h. After this time, infections were either processed for NanoLuc luciferase or visualized and FFUs counted as described for infectivity titration. Percentage neutralization was calculated through comparison of NanoLuc luciferase signal, or FFU counts, to the mean NanoLuc luciferase signal or FFU count of the control in triplicates.

### Treatment with DAAs and inhibitors

To evaluate the quantification of CypA inhibition and the effect of sofosbuvir and molnupiravir on NrHV by NanoLuc luciferase, IVT RNA of NrHV-HiBiT-SN was electroporated into McA-RH7777.hi cells. Following electroporation, 7,500 cells were seeded per well into a laminin-coated 96-well plate. Alisporivir was serially diluted to concentrations ranging from 50 µM to 8 nM, while sofosbuvir and molnupiravir were diluted to concentrations ranging from 100 µM to 0.1 µM and 500 µM to 1 µM, respectively. DMSO-only controls were prepared by mimicking the preparation of the lowest dilution of alisporivir, sofosbuvir, and molnupiravir, adding only DMSO. All dilutions were prepared in DMEM supplemented with 3% FBS, 100 U/mL penicillin, and 100 µg/mL streptomycin (Pen Strep: Sigma-Aldrich). The diluted compounds were added in triplicate 24 h post-electroporation. Cells were incubated at 37°C, 5% CO_2_, for 48 h, after which fresh dilutions of the compounds were added and incubation continued for an additional 48 h. Infections were then quantified using NanoLuc luciferase.

To assess the effect of these compounds on cell viability, McA-RH7777.hi cells were mock electroporated and treated with compounds as described above, with 7,500 cells seeded per well into a laminin-coated white opaque 96-well cell culture plate. Cell viability was then assessed using the CellTiter-Glo luminescent cell viability assay (Promega) following the manufacturer’s instructions and using a CLARIOstar luminometer (BMG Labtech).

### Statistical analyses and multiple alignment

Multiple alignment of the protein sequence of NS5A of HCV genotype 1a (H77, GenBank accession no. AF009606), HCV genotype 2a (JFH-1, GenBank accession no. AB047639), HCV genotype 3a (S52, GenBank accession no. GU814264), HCV genotype 4a (ED43, GenBank accession no. GU814265), RHV-rn1 (GenBank accession no. KX905133), and NrHV-K (GenBank accession no. PV553238) was generated using the MAFFT version 7 web server using default settings, L-INS-i strategy, and the BLOSUM45 scoring matrix (27). Two-way ANOVA, Šídák’s multiple comparisons tests, Dunnett’s multiple comparisons tests, and non-linear regression analyses were performed using GraphPad Prism 10.5.0.

## Data Availability

The NrHV-HiBiT-SN reporter virus sequence with the HiBiT tag inserted into NS5A and carrying the two compensatory SN mutations, but absent previously identified cell culture adaptive mutations, was submitted to GenBank with accession number PZ005968.

## SUPPLEMENTAL MATERIAL

Table S1 jvi.01943-25-s0001.docx Sequences of oligonucleotides.

ASM does not own the copyrights to Supplemental Material that may be linked to, or accessed through, an article. The authors have granted ASM a non-exclusive, world-wide license to publish the Supplemental Material files. Please contact the corresponding author directly for reuse.
