# Supplementary material for: Development of a Norway rat hepacivirus reporter for high-throughput quantification of neutralizing antibodies
Source: J Virol. 2026 Mar 31;100(4):e01943-25. doi: 10.1128/jvi.01943-25 (PMC13098260; doi:10.1128/jvi.01943-25)
Supplement: Table S1 — Sequences of oligonucleotides. [file jvi.01943-25-s0001.docx]

**Appendices**

**Table A1.** Sequences of oligo nucleotides.

| **Primer name** | **Direction (F/R)** | **Oligo sequence** |
| --- | --- | --- |
| **Insertion of FLuc into N-terminus of core** | | |
| TS-O-01253 | F | TACGACTCACTATAGACCATCAC |
| TS-O-01254 | R | AGCCTGCTTCAGCAGGCTGAAGTTAGTAGCTCCGCTTCCCAATTTGGACTTTCCCCCCTTC |
| TS-O-01255 | F | CTGCTGAAGCAGGCTGGAGACGTGGAGGAGAACCCTGGACCTATGGCTTGCAATCTCTTTTTCAACTTC |
| TS-O-01256 | R | CTATAGTGAGTCGTATTATAAGTATC |
| TS-O-01283 | F | CTAACTTCAGCCTGCTGAAGCAGGCTGGAGACG |
| TS-O-00260 | R | AGGTAGCAAGCTCGTCTAGGG |
| **Insertion of *Rattus norvegicus* ubiquitin into NrHV-FLuc-core reporter** | | |
| IDT_Ubi_BuildingBlock | / | GAGAACCCTGGACCTCTGCAGCAAATTTTCGTGAAGACCCTGACGGGCAAGACCATCACTCTTGAGGTCGAGCCCAGTGACACCATCGAGAATGTCAAGGCCAAGATCCAAGACAAGGAAGGCATCCCACCTGACCAGCAGAGGCTGATATTCGCGGGCAAACAGCTGGAGGATGGCCGCACCCTGTCCGACTACAACATCCAGAAAGAGTCCACCTTGCACCTGGTGCTGCGTCTCCGCGGTGGAATGGCTTGCAATCTC |
| TS-O-01425 | F | ATGGCTTGCAATCTCTTTTTCAACTTCCCTTCCACAAAAAAAAACC |
| TS-O-01426 | R | AGGTCCAGGGTTCTCCTCCACG |
| **Insertion of FLuc or eGFP between p7 and NS2** | | |
| TS-O-01221 | F | GAGAACCCTGGACCTGAAGGCGGTGAGTTGCGG |
| TS-O-01222 | R | ACCGCCTTCGTAAGCCTCAACCACATCTGCCAG |
| TS-O-01223 | F | GCTTACGAAGGCGGTATGGAAGATGCCAAAAACATCAAGAAAGG |
| TS-O-01224 | R | AGGTCCAGGGTTCTCCTCCACGTCTCCAGCCTGCTTCAGCAGGCTGAAGTTAGTAGCCAATTTGGACTTTCCCCCCTTCTTG |
| TS-O-01227 | F | GCTTACGAAGGCGGTATGGTGAGCAAGGGCGAGG |
| TS-O-01228 | R | AGGTCCAGGGTTCTCCTCCACGTCTCCAGCCTGCTTCAGCAGGCTGAAGTTAGTAGCCTTGTACAGCTCGTCCATGCCG |
| **Insertion of FLuc or eGFP between duplicated NS5A/NS5B cleavage sites** | | |
| TS-O-00889 | F | TCGTACTCATGGTCGGTTCCTC |
| TS-O-00890 | R | CCAAGAACAGTCGGTCCATGATCC |
| TS-O-01098 | F | ACCGACTGTTCTTGGATGGAAGATGCCAAAAACATC |
| TS-O-01099 | R | CGACCATGAGTACGACCAGCTGCAATCCGTCCAGCTGCCGCTCAATTTGGACTTTCCC |
| TS-O-00305 | F | TCTGGATCATGGACCGACTGTTCTTGGATGGTGAGCAAGGGCGAGGAGCTG |
| TS-O-00306 | R | ACCAATTGAGGAACCGACCATGAGTACGACCAGCTGCAATCCGTCCAGCTGCCGCTCTTGTACAGCTCGTCCATGCCGAG |
| TS-O-01105 | F | GGCATCCCGTGTCGGTTTGGTACAAGG |
| TS-O-01106 | R | CCAAGAACAGTCGGCCCATGATCCAGACGACTG |
| TS-O-01184 | F | GCGGCAGCTGGGCGGATTGCAGCTGGTCGTACTCATGG |
| TS-O-01185 | R | CCATGAGTACGACCAGCTGCAATCCGCCCAGCTGCCGC |
| **Generation of HiBiT NrHV reporters** | | |
| TS-O-01591 | F | GCTGTTCAAGAAGATTAGCGGGAGTTCTGGCTGCAATCTCTTTTTCAACTTCCCTTCC |
| TS-O-01592 | R | ATCTTCTTGAACAGCCGCCAGCCGCTCACAGCCATAGATGGTTTACAGCGGAAACG |
| TS-O-01593 | F | ATGGCTTGCAATCTCTTTTTCAACT |
| TS-O-01594 | R | AATCTTCTTGAACAGCCGCCAGCCGCTCACGCAAGCCATAGATGGTTTACAGCGGAAAC |
| TS-O-01595 | F | CTGTTCAAGAAGATTAGCGGGAGTTCTGGCCAAATTTTCGTGAAGACCCTGACGG |
| TS-O-01596 | R | GAGATTGCAAGCCATTCCACCGCGGAGACGCA |
| TS-O-01597 | F | CTGTTCAAGAAGATTAGCGGGAGTTCTGGCTACGAAGGCGGTGAGTTGC |
| TS-O-01598 | R | AATCTTCTTGAACAGCCGCCAGCCGCTCACACCGCCTTCGTAAGCCTCAACC |
| TS-O-01599 | F | CGTGAGCGGCTGGCGGCTGTTCAAGAAGATTAGCGTCCGCCAACCAAAACCAAAAC |
| TS-O-01600 | R | CGCCAGCCGCTCACGCCAGAACTCCCCGGAGATTGAGATGGAAGGG |
| **Generation of NrHV-HiBiT-KLSN/SN** | | |
| TS-O-01711 | F | GGACGGAAGCTCCTCGACCACGAGCTCATCC |
| TS-O-01712 | R | TTAGCGGTTTTGGGTTTGGTTGGCGGACG |
| TS-O-01713 | F | TCCTGCTCGCAAAGGCGGGCATCG |
| TS-O-01714 | R | TGAATAGGGCAGTTAGAGAAACAAACTCATCC |
| **Introduction of W2289S mutation to RHV-rn1-HiBiT-SN** | | |
| TS-O-02128 | F | TTAGCGTCCGCCAACCAAACCCAAAACCGCTAAA |
| TS-O-02129 | R | TTTAGCGGTTTTGGGTTTGGTTGGCGGACGCTAA |
| **qPCR primers/probes** | | |
| TS-O-00561 | F | TACATGGCTAAGCAATACGG |
| TS-O-00562 | R | AAGCGCAGCACCAATTCC |
| TS-O-00563 | F | [6-FAM]CTCACGTACATGACGTACGGCATG[BHQ1A-6FAM] |
| **cDNA synthesis** | | |
| TS-O-00319 | R | GCTTCCTGGAGCGGGCTAGATACTG |
| **Full ORF amplification** | | |
| TS-O-00316 | F | TGTCCCACAGCGAGCCTGGGATAAC |
| TS-O-00318 | R | CCAAGCCCCAATGCCGTCCGGCACCGCTGCCCTTTTCGG |
